# Who counts in poverty research?

**DOI:** 10.1177/00380261231213233

**Published:** 2023-11-29

**Authors:** Daniel Edmiston

**Affiliations:** Department of Political Science and Public Law, Institute of Government and Public Policy (IGOP), Autonomous University of Barcelona, Barcelona, Spain

**Keywords:** data coverage, deep poverty, epistemic erasure, non-private-household population, welfare politics

## Abstract

Mainstream poverty analysis currently renders certain people and degrees of privation more socially legible than others across high-income countries. This article examines how these hierarchies carry through to and corrupt wider social scientific analysis, inscribing differential value to actors and phenomena in ways that undermine social understanding and explanation. First, conventional approaches to poverty analysis and measurement obscure the *de facto* prevalence of deep poverty, as well as those most subject to its violence. Second, a growing number of hyper-marginalised groups are missing from population income surveys, undermining the accuracy of (deep) poverty estimates and public understanding of both its determinants and dynamics. Third, the inferential and external validity of income surveys is significantly diminished by problems surrounding data quality and coverage. Attempts to address this have principally focused on improving data quality, but as demonstrated in this article, these strategies exacerbate poor representation of the lowest-income groups in distributional analysis. Much more than merely technical or pragmatic, these are theoretical and normative judgements about who counts in welfare policy and politics. Overall, I demonstrate how current data practices occlude some the most violent forms of denigration and exploitation that structure advanced marginality, particularly the gendered, racialised, bordering and ableist practices underpinning state–citizen dynamics. Focusing principally on the UK context, I argue that the epistemic erasure committed features in and systematises a policy blindness to deep poverty for some of the most marginalised social groups making it harder to evidence its effects and address its causes across high-income countries.

## Introduction

Since the 2007/8 global financial crisis, empirical sociology has made great headway to identify the varying extremes of economic advantage domestically and globally. This ‘return of inequality’ has motivated invaluable methodological innovation and experimentation to better understand economic elites and their bearing on social fragmentation (Savage, 2021). In many ways, this body of work offers a necessary reckoning with our ‘blind spots’ across the social sciences and reflects growing recognition of the need to ‘look up’ to those people and places we know relatively little about (Edmiston, 2018; Paton, 2018). By contrast, less concerted, coordinated effort has been made to advance research methods and understanding of marginality in the lowest ranges of the socioeconomic order in recent years (Bramley & Fitzpatrick, 2023; Edmiston, 2022; Varner et al., 2017). Of course, this is the exception to the rule if we take a broader view of the social sciences. Historically, the sociological gaze has tended ‘downwards’ towards those experiencing privation, often neglecting those actors and institutions that have the greatest bearing on low-income dynamics. Today though, attempts to evidence the changing nature and severity of poverty in high-income countries have become somewhat fragmented (Crossley et al., 2019). In part, this stems from a (misplaced) belief that the incidence of destitution is negligible: reserved for ‘other’ people and places outside of Europe and North America (cf. Gaisbauer et al., 2019). However, it also stems from the problems encountered when researching extreme marginality: that is, little consensus on the appropriate terms of reference in mainstream poverty analysis, as well as systematic challenges surrounding data quality, coverage and measurement. By mainstream poverty analysis, I refer to official poverty statistics, but also to the dominant data practices, methods and infrastructure that mediate ways of knowing, quantifying and responding to poverty across the social sciences.

Whilst recognition of the need to better understand the changing severity of poverty in high-income countries has been relatively slow and segmented, it has nonetheless gathered pace in recent years (Alston, 2018, p. 15; Social Metrics Commission [SMC], 2020). Specifically, there has been growing concern that mainstream poverty analysis and government reporting on low incomes is failing to capture the living standards of the lowest-income groups worst affected by socioeconomic upheaval and welfare state recalibration across late capitalist contexts (Edmiston et al., 2022a). Recent work that attempts to address this has proven instrumental for highlighting some of the shortcomings with contemporary poverty research internationally (Gaisbauer et al., 2019; Jolly et al., 2022). However, conceptual and methodological advancements across distinct sub-fields often remain siloed off from one another; inadequately integrated to effectively scrutinise the social politics underpinning economic marginality (Du Toit, 2009). In response, this article demonstrates how a failure of distinct epistemic communities to engage with and learn from one another is stunting social scientific analysis of disadvantage, both analytically and empirically. In the US, for example, Brady (2022) illustrates how stratification research is often hampered by definitional and measurement problems when it comes to establishing the incidence, effects and (racialised) dynamics of poverty. In the UK, the Office for Statistics Regulation (OSR) recently undertook a review of income-based poverty statistics and concluded there was an urgent need to address the coherence, quality and coverage of mainstream distributional analysis to better understand and respond to ‘those in deep poverty’ (OSR, 2021, pp. 15–18; see also Department for Work and Pensions [DWP], 2022a).

For those seeking to do so, however, a series of problems arise that currently compromise effective quantification of poverty prevalence, dynamics and determinants. Focusing principally on the UK context, this article considers three shortcomings associated with mainstream approaches to poverty analysis to demonstrate how existing data practices generate hierarchies of knowledge that count, measure and value some low-income populations more than others. I argue that these hierarchies carry through to and corrupt wider social scientific analysis, inscribing differential value to actors and phenomena in the research and policymaking process, in ways that undermine social understanding and explanation. Failing to address this means sociological analysis risks reproducing the very exclusions and symbolic misrecognition implicated in disadvantage. Reflecting on the epistemic and ontological significance of this, I illustrate how mainstream poverty analysis is a system of classification in a number of important ways (Tyler, 2015), but it is also a site of effacement: of particular people, forms of privation and the dynamics that give shape and legitimation to advanced marginality. I argue that it is this, as much as systems of classification, that makes a ‘biopolitics of disposability’ – and perhaps its most violent technologies – possible. This article outlines an agenda for researching poverty that has stronger diagnostic purchase to account for the full extent and character of deepening inequalities. I argue for an integrated approach to distributional analysis that would animate more effective forms of sociological analysis, better equipped to examine the gendered, racialised, bordered and ableist dynamics underpinning class (dis)advantage and the deepest forms of poverty across high-income countries.

### The social violence of parsimony in mainstream poverty analysis

This article examines three interlinked issues undermining our empirical understanding of poverty and does so to reflect on the significance of mainstream data practices that determine the legibility of extreme marginality and its governance across high-income countries. First, conventional approaches to poverty measurement and analysis currently gloss over definitional and measurement questions guiding attempts to establish the changing extent of ‘deep poverty’ and whether it is a distinct, meaningful category of experience. Second, current data practices reify judgements about *who counts* when it comes to researching poverty and the policies conceived to tackle it. Whilst those living outside of private households are often part of the inferential population in welfare politics, they are not part of the target population and thus sampling frame of income surveys underpinning official statistics and mainstream poverty analysis. Third, the inferential and external validity of income surveys is compromised by problems of poor data quality and coverage. Thus far, attempts to address this have principally focused on improving data quality, but as demonstrated in this article, these strategies risk amplifying poor representation of the lowest-income groups in distributional analyses.

In certain respects, these shortcomings are not new. Over time, there have been important conceptual and applied innovations to address issues of measurement (e.g. Gordon & Townsend, 1990; Hick, 2015), representation (e.g. Carr-Hill, 2015) and data quality (e.g. Brewer et al., 2017). However, these advances have tended to develop quite separately across distinct subfields, in a manner that stifles the descriptive and explanatory purchase of research on disadvantage. A consequence is ‘apartheid in the research community’ (Jenkins, 1991, p. 462) with poverty researchers often talking past or over one another, focused on their respective explanatory frameworks without reflecting on the broader sociological significance of the poverty research landscape and their own role within it. Against this backdrop, a fragmented evidence base suggests the depth of poverty is increasing across many high-income countries with mainstream poverty analysis increasingly out of step with, and poorly suited to capturing these trends (Edin & Shaefer, 2015; Edmiston, 2022). Insights from such work have tended to take on an ‘essentially supplementary and illustrative role in accounts of poverty’ for the sake of parsimony in government reporting and academic analyses of low incomes, with qualitative research findings often under-appreciated and side-lined in typical hierarchies of evidence (Du Toit, 2009, p. 238). As a result, dominant ways of conceptualising and measuring poverty tend to be inadequately equipped to make the full causes and dynamics of disadvantage intelligible.

What are the consequences of failing to translate what is scientifically known, at least within distinct epistemic communities, into that which is socially known and responded to? In many ways, it has lead to further fragmentation in the field of poverty studies where ‘new stories are being told, traditional approaches have apparently failed, and new poverties are emerging’ (Crossley et al., 2019, p. 8). Without shared understanding of ‘the problem’ and how to measure it, this retreat into epistemic relativism means those worst affected by the contradictions of global financial capitalism are less empirically and analytically visible in the research and policymaking process across high-income countries. This underlines a Janus-faced tendency across high-income countries to simultaneously obsess over and omit those worst served through urban transformation, socioeconomic restructuring and welfare state recalibration in the wider sociocultural and political imaginary. The poorest are often subject to considerable, invasive state intervention and surveillance that problematises behaviours and pathologises negative social outcomes (Dagdeviren et al., 2017). Either through disembodied data-driven surveillance or more routine monitoring practices to allocate or withhold assistance, such forms of surveillance anticipate and establish distinctions between the ‘deserving’ and ‘undeserving’ poor (Mayblin, 2019; Shildrick & MacDonald, 2013). These methods of surveillance are often justified as a necessary means through which to understand and tackle social exclusion. And yet, many of those at the centre of these interventions are missing or rendered invisible through official statistics and mainstream poverty analysis.

Focused attention on those missing or obscured helps recast our understanding of phenomena that are present and visible, the processes by which this is made possible and the political agendas this serves. Here, the ‘practices of silence, invisibility and emptiness’ created through mainstream poverty analysis can come to be recognised ‘respectively as voluble, conspicuous and potent’ (Scott, 2018, p. 6), truncating understanding of social (dis)advantage and implicated in its reproduction. As I will demonstrate, the majority of distributional analysis in high-income countries re-marginalises the privation of certain social groups who are already poorly served through the ‘government of poverty’ (Du Toit, 2009). With particular groups and degrees of privation coming to count for less in distributional analysis, I demonstrate how this delimits wider social scientific analysis in ways that risk occluding some of the more violent forms of denigration and exploitation that structure marginality, particularly the gendered, racialised, bordering and ableist practices underpinning state–citizen dynamics. The following sections of this article detail how this occurs across three key areas, before reflecting on what this means for sociological analysis and its bearing on the governance of (advanced) marginality.

### Getting the measure of ‘deep poverty’

Debates about the relative merits of different approaches to poverty measurement have long highlighted limitations associated with threshold measures of low income. The most central concern being that threshold indicators measure changing *rates* of poverty but tell us little about the intensity of privation (Hirsch et al., 2020). In response, there has been growing, but fragmented interest in the problem of ‘deep poverty’ across high-income countries in recent years with a range of measures emerging to explore the low-income dynamics and living standards of those falling, to varying degrees, below relative poverty lines (Brady & Parolin, 2020; Edmiston, 2022; OSR, 2021).

However, there is little consensus on how the depth of poverty should be conceptualised or measured – in particular, whether ‘deep poverty’ should be understood as an *absolute* or *relative* condition. Whilst both ways of thinking about deep poverty are prevalent, there is a tendency to characterise it as reflecting a more *absolute* condition of privation (e.g. OSR, 2021, p. 14). It is questionable though whether any such conception of poverty (in the absolute sense) could ever be universal *and* meaningful; that is, understood and defined independently of other conditions, people or things. Even severe material deprivation indicators are subject to prevalence weighting to account for their contingent significance (DWP, 2020). Arguably, it is more productive to conceptualise (deep) poverty as a continuum of disadvantage, with depth implying location – the social and material distance one lies from a given standard. Other terms such as ‘extreme’ or ‘severe’ poverty suggest that the wider category of poverty is not, by its nature, ‘extreme’ or ‘severe’ in the resources available to low-income households and the social violence this inflicts. There is therefore an evaluative component to ‘extreme’ or ‘severe’ poverty that confounds normative appraisal with the conceptualisation and measurement of poverty. ‘Deep poverty’ by contrast can be understood as a *relative* condition: experienced and defined in reference to a higher material standard of privation.

When it comes to measurement, though, questions remain about what makes deeper forms of poverty distinguishable from shallower forms. Is it the absolute or relative distance one falls below a given standard that matters most? Being towards the very bottom of an income distribution? Or experiencing (greater degrees of) material deprivation? Current measures adopted by research analysts and campaigners in the UK prioritise one, some or all these ways of understanding deep poverty, drawing on both direct and indirect indicators of living standards. For example, a range of indicators and terms are currently in circulation that either draw on (a) income-based measures, e.g. the ‘poverty gap’ (OECD, 2022), ‘low-income gap’ (Hirsch et al., 2020), ‘depth of low income’ (Padley & Stone, 2022), ‘deep poverty’ (Joseph Rowntree Foundation [JRF], 2022; Lee, 2020; SMC, 2020) and ‘very deep poverty’ (JRF, 2022); (b) composite measures of income *or* material deprivation, e.g. ‘destitution’ (Fitzpatrick et al., 2020); or (c) composite measures of income *and* material deprivation items, e.g. ‘severe poverty’ (Fitzpatrick et al., 2020), and ‘severe low income’ (DWP, 2020). According to each of these plausible ways of conceptualising and measuring it, the extent, nature and demographic composition of deep poverty changes.

**Figure 1:**
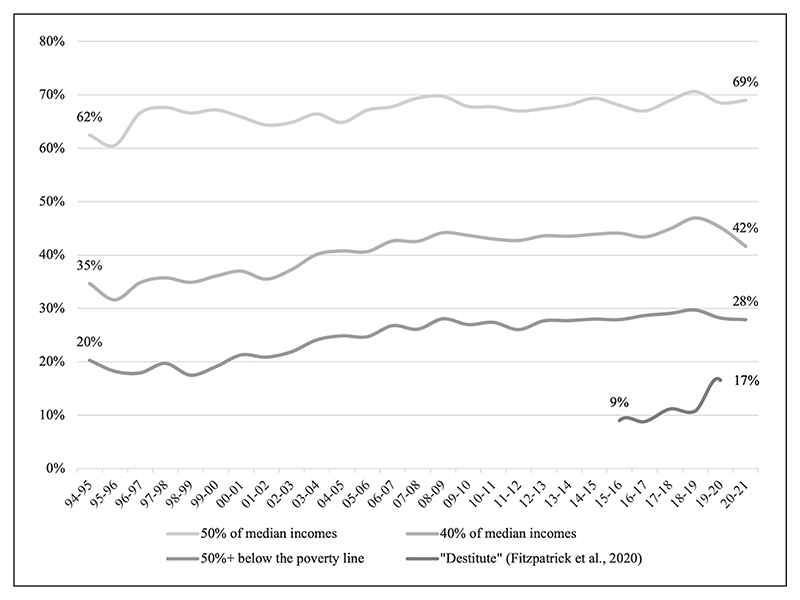
Proportion of the low-income population (below 60% of median incomes) in ‘deep poverty’ according to different measures, 1994–2021. Source: DWP (2022b), author’s calculation.

Figure 1 demonstrates how the incidence of deep poverty has increased over the last 25 years in the UK but depending on the measure chosen, prevalence varies considerably: ranging from as many as 9.3 million people (falling below 50% of median incomes) to as low as 2.4 million people (in destitution) in 2020-21. Overall though, those in deeper forms of poverty now make up a larger share of the low-income distribution than they did a quarter of a century ago. In 1994, 20% of those in relative poverty had an income that fell more than 50% below the poverty line but by 2021 this had risen to 28%. Over the same time period, the proportion of those in relative poverty falling more than 40% below median incomes increased from 35% to 42%. These trends are not currently captured by the headline indicators typically employed in government reporting of low incomes across high-income countries (Edmiston, 2022).

As expected, different measures of deep poverty also entail varying degrees of hardship with the average incomes of different categories falling, and the prevalence of (severe) food insecurity increasing, further down the income distribution (Table 1). The median disposable income of someone in poverty according to the government’s main measure (below 60% of median incomes) is 74% of the relative poverty threshold, but as low as 38% for someone falling more than 50% below the poverty line. Two people with these respective incomes would both be categorised as falling below the relative poverty threshold but would radically differ in terms of the nature and degree of hardship experienced.

Depending on the measure chosen, different people also emerge as a policy priority for government intervention and support. An extensive body of work has analysed the relationship between markers of social difference and material resources to establish the disproportionate exposure of certain groups to the risks of poverty. For example, women (23%), children (31%), Black, Asian and minority ethnic (BAME) people (38%) and those experiencing a limiting health condition or disability (27%) are all more likely to be in relative poverty than the wider general population in the UK (22%) (Table 2). However, looking at the demographic composition of those experiencing varying degrees of financial hardship suggests certain groups are more at risk of deep poverty than others. For example, single childless households, men, unemployed people, BAME communities and those aged 16–24 and 55–64 make up a disproportionate share of those falling more than 50% below the poverty line in the UK (Table 2). Accounting for uneven dispersion below the poverty line has the capacity to offer fuller insight into the unequal effects of welfare reform over time. As receipt of working-age social transfers has become increasingly conditional on fulfilling work-related requirements or (albeit partially and temporarily) on child-rearing activities, the risk of (deep) poverty has increased considerably for single childless households, particularly unemployed single men. Looking at the changing profile and composition of poverty, the same trend is observed in the US context (Varner et al., 2017), underlining some of the gendered dimensions to welfare state recalibration that are often occluded through headline indicators and government reporting of low incomes.

By its very nature, any unitary definition or measure of poverty adopted comes to classify and categorise the social world, making certain people and forms of privation more readily visible than others in distributional analysis. As demonstrated above, those experiencing the most profound forms of hardship are often obscured through a limited number of partial indicators of low income in mainstream poverty analysis. Reflecting on how these indicators are then taken up by and reproduced more broadly across the social sciences, it is possible to see how mainstream poverty analysis does not just create and reproduce social knowledge, it also procures legitimacy for the production and circulation of certain knowledge claims that, in turn, set the parameters and possibilities for state (in)action and accountability.

To maximise the construct and criterion validity of any poverty measure, the cut-off, indicator or threshold adopted has to be theoretically motivated and empirically driven. Failing to ensure this, risks reifying social categories that obscure the realities and circumstance of those falling deeper into poverty as well as the state-citizen dynamics that make the most extreme forms of marginality possible. Of course, the indeterminate nature of income thresholds and contingency of social outcomes across population sub-groups make it almost impossible to identify any singular indicator, but there is nonetheless a need to pursue programmes of research that centre *a posteriori* poverty definition and measurement (e.g. Gordon & Townsend, 1990). Such approaches would engender greater clarity around what is being shown in distributional analysis and why it matters. This is not to assume that we can or should arrive at a unitary definition or measure of deep poverty. Even an empirically informed threshold must sit alongside a plurality of measures to account for the changing profile of poverty and its nature as a corrosive social relation (Lister, 2021). And in this respect, the Foster–Greer–Thorbecke (FGT) Index has proven particularly instructive in accounting for diversity and intensity within and between population subgroups. Capturing inequality and the relative impact of economic growth and policy interventions on poverty reduction, the FGT Index has principally been developed and deployed by welfare economists looking to ‘help low-income countries assess and combat poverty’ (Foster et al., 2010, p. 515). However, it could be more widely deployed in high-income countries to examine social outcomes and living standards associated with varying degrees of financial hardship across population subgroups.

### Who counts in official statistics and mainstream poverty analysis?

Official statistics measure, but also create governable populations of value to national interests and political objectives. The ‘rise of statistical thinking’ has offered a means through which to aggregate information on people, and design public policy according to the social categories deemed relevant or strategically necessary (Ruppert & Scheel, 2021). Understood in this way, publicly funded surveys delimit ways of ‘knowing’ and ‘unknowing’ populations: generating possibilities for the erasure or visibility of social phenomena, forming the basis for (welfare) state disavowal or intervention. Across high-income countries, income surveys funded by national governments that underpin main-stream poverty analysis are no different, and typically identify private households as their target population. As a result, those not living in private households are currently excluded from the sampling frame of population income surveys and, in turn, the distributional analyses undertaken to examine unequal social transformation. Despite this, inferences are routinely drawn from income surveys and generalised to the wider general public in social scientific analysis. One might think that such category slippage is tolerable given that the population subgroups missing from income surveys are relatively small and thus unlikely to have much bearing on poverty prevalence, trends or determinants. However, there are several reasons why this ‘missing minority’ must be recognised as a crucial omission in poverty statistics and analysis.

First, the size of the ‘missing minority’ is non-trivial, covering a wide range of people: from homeless and displaced populations to those residing in care homes, hospitals, military accommodation and immigration removal centres. By design, these groups are missing from income surveys underpinning (supra-)national statistics and mainstream poverty analysis. Income surveys also tend to under-represent those in unstable or multiple occupancy households such as migrants and transient populations (Junes, 2022; Schanze, 2021). Across Europe, at least 6.6 million people are not living in private households (Eurostat, 2023). This is equivalent to the combined population of Denmark and Cyprus. The average non-coverage rate of the non-private-household population in Europe is 1.5%, varying considerably from as high as 5.1% in Sweden to as low as 0.5% in Spain (Eurostat, 2023). In Canada, just under 2% of the population are missing from official poverty statistics, including those living on Indigenous settlements, reserves and extremely remote areas. This rises to as high as 3% in Australia. In the UK, between 1.5% and 2% of the population are understood to be living outside of private households and thus missing from distributional analyses (Bramley et al., 2018, p. 65).^3^ Crucially, this ‘missing minority’ is growing much faster than the private household population typically sampled across high-income countries (Schanze, 2021).

Second, the exclusion and under-representation of such groups matters to the extent that they differ from the wider population and there is evidence to suggest that those missing perform much worse in terms of their social outcomes and living standards (e.g. Fitzpatrick et al., 2020; Gaisbauer et al., 2019). Specifically, those population subgroups currently missing are much more likely to ‘display serious levels of poverty, including destitution and low well-being’ (Bramley et al., 2018, p. 4). Carr-Hill (2015, p. 255) estimates that ‘250 million of the poorest’ individuals are currently missing from global income surveys which significantly compromises our capacity to accurately estimate the extent of (deep) poverty, as well as who is most at risk. For example, Nicaise et al. (2019) incorporate three population subgroups (homeless people, undocumented migrants and travellers) currently outside the sampling frame of income surveys in Belgium, and based on their surveyed characteristics estimate that the official poverty rate is 0.6–1.7 percentage points higher than initially thought. Whilst deep poverty estimates are also likely to be much more pronounced given the living standards of those sampled, incorporation of the wider non-private-household population would likely yield a more complex picture given the variable incidence of poverty amongst different subgroups.

That said, qualitative research on financial hardship illustrates how hyper-marginalised groups on the periphery of institutional recognition and support – such as those affected by homelessness or No Recourse to Public Funds – are often poorly accounted for, or missing, in distributional analyses and policy evaluation (e.g. Edin & Shaefer, 2015; Jolly et al., 2022; Pinter et al., 2020). If a considerable number of this ‘missing minority’ are more likely to experience (deep) poverty – and there are reasonable grounds to believe that they do – non-coverage rates relative to the whole population may initially appear minor, but as a proportion of the low-income population these gaps in data become cumulatively quite significant.

To what extent does the exclusion of this ‘missing minority’ skew poverty analysis and in turn compromise effective anti-poverty policymaking? The answer depends not only on the size and living standards of the non-private-household population but also their demographic characteristics. Across many high-income countries, there is evidence to suggest that those missing from income surveys are more likely to suffer from multiple, compounding disadvantages over the life course (Junes, 2022; Nicaise et al., 2019; Office for National Statistics [ONS], 2015). To take the UK as an example, those with complex support needs, ‘migrants with limited English, people without or losing work, households on or applying for UC [Universal Credit], people with mental or physical health problems, renters and people not in private households’ are both considerably more likely to experience destitution and under-represented in income surveys (Bramley & Fitzpatrick, 2023, p. 21). The latest available data also suggest those residing in communal establishments are much more likely to be migrants, economically inactive, BAME and not have any formal qualifications (ONS, 2015). Elderly women, many with limiting health conditions and disabilities, are the largest demographic group under-represented in official poverty statistics given their disproportionate residence in institutional care homes (Corlett et al., 2018). And at least amongst the working-age population, men make up a larger share of the non-private-household population because they are more likely to experience homelessness, be in prison or the armed forces (Corlett et al., 2018, p. 50).

Depending on their gender, ethnicity, disability, citizenship status and life course stage then, key demographics are more likely to be concentrated in certain types of communal establishment or living situations. Analytically, the meaning of low incomes will change between such groups according to the distinct environments they find themselves in and whether their needs are met within such contexts. Incorporation of this ‘missing minority’ has the capacity to nuance poverty analysis with incomes often considered necessary but not sufficient to guarantee attainment of living standards. However, in many communal establishments, incomes may not even be necessary which stands to complicate established theories of poverty and place. Those currently outside the sampling frame of income surveys are often the focus of considerable, often intensive, (welfare) state intervention but are not currently reflected in official poverty statistics. This makes it difficult to effectively assess state–citizen dynamics and the *relative* impact of public services on the non-private-household population. This matters because a considerable part of welfare politics and policy is about moving people out of the types of situations (e.g. homelessness) that also determine whether or not they are counted in poverty analysis. Low-income dynamics mean some people will not only move in and out of poverty, but also the sampling categories that dictate whether their financial situation is reflected in official statistics.

Overall, existing data infrastructure and practices reproduce gaps in knowledge about many of the key populations that demand most urgent attention from researchers and policymakers. The exclusions made possible through current sampling decisions systematically under-represent key demographics from the counting process. Such data practices demonstrate the inherently political nature of survey statistics given their capacity to make and unmake social groupings ‘as a knowable object of government’ (Ruppert & Scheel, 2021, p. 4). At present, mainstream poverty analysis tends to reify particular formations and accounts of poverty in social scientific analysis, whilst dissolving others in public consciousness. To deem this insignificant renders those currently outside the sampling frame of population income surveys analytically invisible in the wider sociocultural and political imaginary. It also reinforces stylised notions of what constitutes a social problem worthy of attention through prescribed units of analysis and welfare state (dis) intervention. The epistemic erasure committed features in and systematises a blindness to the privation experienced by many of the most marginalised social groups, undermining social understanding and explanation of advanced marginality across high-income countries.

As demonstrated, incorporating the non-private-household population into distributional analysis would reframe our interpretation of ‘progress’ towards tackling financial hardship across late capitalist contexts.^4^ The most costly but effective way of achieving this would be to expand the target sample of population income surveys to include the non-private-household population. In the UK, a scoping study drawing on census-based estimates demonstrates the insights this could offer, but government agencies responsible for official poverty statistics are yet to take this forward (Bramley et al., 2018; OSR, 2021). An alternative is to draw on insights from qualitative studies and specialist surveys of the non-private-household population to correct for non-coverage error in distributional analyses. Integrating surveys that cover many of those typically ‘missing’ and under-represented in conventional datasets, Bramley and Fitzpatrick (2023, pp. 8–9) develop calibrated models that ‘overcome the limitations of mainstream household surveys to investigate the incidence, risks and drivers of destitution’. To fully account for diversity within the non-private-household population though and their distinctive bearing on poverty estimation, systematic collection and compilation of data on the living standards of population subgroups are needed (Schanze, 2021). Such datasets could then be used to impute the living standards of the non-private-household population and use these parameters to address the exclusion of diverse population subgroups in mainstream poverty analysis.

### Building on sand: Poor data coverage and quality

**Figure 2:**
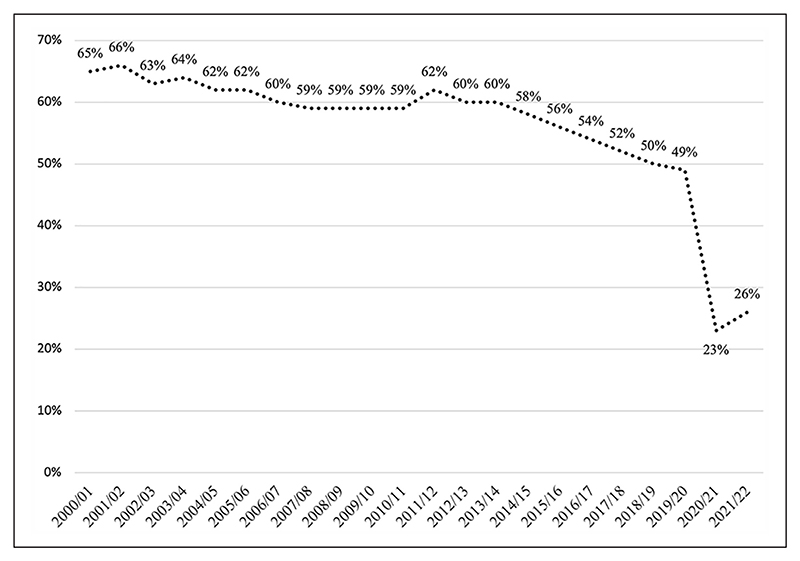
Response rate to the Family Resources Survey 2000–2021.^6^

National income surveys are foundational to social and public policy: they are intended to provide aggregated information on the outcomes and living standards of sampled populations to inform effective policymaking and public action. However, methodological advances have unearthed problems with the quality and coverage of data available on low-income populations. For example, recent work linking longitudinal surveys to administrative data in the US raises serious concerns about the reliability of poverty estimates given systematic errors in reported incomes and benefit receipt (Meyer et al., 2021). To explore the significance of this, I focus here on the Family Resources Survey (FRS) in the UK context (DWP, 2022c), to illustrate how the current approach to and treatment of household income surveys can place poverty analysis and policy formulation on shaky ground. In terms of data coverage, the FRS is the primary data source for official poverty statistics in the UK but suffers from a declining response rate and biases in non-response, undermining the inferential and external validity of distributional analysis. Over the last 20 years, the response rate to the FRS has fallen considerably: from 65% in 2000/1 to just 49% in 2019/20 (DWP, 2022c) (Figure 2). Whilst the Department for Work and Pensions^5^ acknowledges the growing ‘risk of systematic bias in survey results’, they nonetheless suggest that the response rate is not ‘unreasonable’ given the size and complexity of the FRS (DWP, 2022c, n.p.). Despite distinctive sampling and recruitment strategies, it is clear the UK fares much worse than its international counterparts (Figure 1). According to the latest available data, the Canadian Income Survey (80%), the Australian Survey of Income and Housing (74%), the US Current Population Survey (73%) and domestic surveys underpinning EU Statistics on Income and Living Conditions (EU-SILC) (60% on average) all have much higher response rates. The potential for non-response bias in poverty analysis is considered by the DWP in the UK but accounted for much less systematically than in other contexts such as the US (e.g. Yang et al., 2021). This is significant given that those refusing to take part in FRS are more likely to be: living alone, male, BAME, have fewer children and live in households containing at least one paid working adult (Maher & Tait, 2012, pp. 23–26). However, this understanding of non-respondent characteristics is based on the FRS conducted in 2008/9 when the overall response rate was considerably higher than it is currently. More recent examination of non-response bias has not been published, which may, in part, be explained by a declining completion rate to the non-response form in FRS: falling from 62% in 2005/6 to 47% in 2008/9 (Maher & Tait, 2012, p. 10). That said, those living in accommodation with the lowest Council Tax bands (A–C) are now slightly under-represented in the FRS (DWP, 2022c). These trends give reasonable cause for concern about the extent to which the FRS provides a solid foundation upon which to construct accurate poverty estimates.

In terms of data quality, poor correspondence between income, expenditure and material deprivation indicators amongst those towards the very bottom of the income distribution has ‘long been acknowledged as a problem that has the potential to distort estimates of poverty’ (Saunders & Bradbury, 2006, p. 345; see also Brewer et al., 2017).^7^ This presents a particular challenge for those looking to fulfil or assess (supra-)national policy objectives that ‘leave no-one behind’. Thus far, attempts to address this have tended to focus on either data exclusion or data manipulation.

To manage uncertainty surrounding reported incomes, data producers and users routinely exclude the lowest-income observations from distributional analyses or the modelling of policy effects. Such data practices are considered necessary given that some of the lowest-income respondents report incomes that are considerably lower than their reported expenditure and national social security entitlements (Brewer et al., 2017). In response, many analysts remove the lowest percentiles (1–3%) of the income distribution from distributional analyses, but others have gone further to exclude the lowest income decile altogether from headline indicators (Australian Bureau of Statistics [ABS], 2004, pp. 28–29). Saunders and Bradbury (2006) demonstrate how certain data exclusion practices risk *underestimating* poverty and economic polarisation. This is because there are several plausible explanations for a mismatch between incomes, living standards and national benefit levels amongst low-income households. These include ineligibility, deductions, sanctions and non-take-up of social security benefits. Equally, higher reported expenditure relative to reported incomes may, in part, be a function of debt accumulation, the additional costs associated with poverty, pawning or selling household items to cover living costs, over-reported expenditure, or spending more than is typical during the period surveyed for those towards the bottom (if not 1%) of the income distribution. Table 3 demonstrates how removing the lowest income cases (1–3%) from distributional analysis may be prudent given their reported incomes and characteristics, but such practices risk excluding those experiencing heightened material deprivation (food insecurity) as well as those reporting incomes that may genuinely reflect fluctuations in their earnings (e.g. due to self-employment).

Leaving aside data exclusion, significant headway has been made internationally to improve the accuracy of income data by linking administrative records or imputing likely incomes on the basis of social security, demographic or tax data (e.g. Corlett et al., 2018; Meyer & Mittag, 2019). Research adopting such approaches suggests that the level and intensity of poverty in the US is lower than initially thought with this decreasing over time (Meyer et al., 2021; Parolin & Brady, 2019). Meyer et al. (2021) also find that this reprofiling changes the demographic composition of those living in ‘extreme poverty’: from primarily families with children to single childless individuals. There are, however, risks in drawing inferences from such sources without recognition that linked datasets can introduce new sources of measurement error, and invariably only cover the target sample of income surveys (usually private households) rather than the wider low-income population. Equally, a sole emphasis on the use of administrative records misses those ineligible for, or on the periphery of, social security support such as those with No Recourse to Public Funds or the ‘missing workless’ not claiming benefits (Edmiston et al., 2022b; Shildrick et al., 2012). Indeed, distributional analyses employing ‘improved measures, higher-quality data and several thresholds’ that go some way to addressing these issues find that deeper forms of poverty are likely underestimated, particularly when accounting for homeless populations (Brady & Parolin, 2020, p. 2338).

Across high-income countries, researchers have sought to correct for measurement error in household income data by imputing likely income levels or benefit receipt based on respondent characteristics (Corlett et al., 2018; Meyer et al., 2009). For example, there is growing concern about an increasing ‘expenditure gap’ between administrative records on social security spending and those currently captured in microdata releases in the UK and the US (Meyer et al., 2009; OSR, 2021). Recent work suggests that almost a ‘fifth [£44 billion] of benefit spending is missing from the best source of household income data’ and that this has grown considerably over the last 20 years in the UK (Corlett et al., 2018, p. 44). In light of this, Corlett et al. (2018) develop an imputation method that allocates this ‘missing’ expenditure to FRS respondents not reporting (enough) benefit receipt based on their characteristics, employment status and location. Assuming this models a truer estimation of the income distribution, the authors conclude that the *de facto* incidence of poverty is considerably lower than official poverty statistics suggest. The findings of this analysis have proven highly influential, leading the Office for Statistics Regulation to recommend addressing ‘under-reporting at the bottom end of the income distribution’ as a matter of priority (OSR, 2021, p. 22).^8^ By their own admission though, the authors base their analysis on ‘some major simplifications and assumptions’ (Corlett et al., 2018, p. 60) that bring into question such imputation methods in income surveys.

First, the authors characterise the gap between reported receipt in FRS and official government records as squarely an issue of measurement error. As a result, they fail to fully account for how non-response bias and non-coverage error can go some way to explain the growing ‘expenditure gap’. Given the collapsing response rate to FRS and higher incidence of poverty amongst the non-private-household population (Junes, 2022; Schanze, 2021), it is likely that a greater proportion of outturn benefit spending goes towards those missing or under-represented in FRS than is currently accounted for. Expenditure gaps being highest for those affected by limiting health conditions, disabilities and old age also gives a clue as to the extent to which benefit spending may be going to communal establishments and thus not captured by FRS (Corlett et al., 2018, pp. 52–56). Second, the imputation method adopted treats the ‘expenditure gap’ as largely driven by those towards the bottom of the income distribution under-reporting benefits (Celhay et al., 2022, p. 16; Corlett et al., 2018). Whilst there are good reasons to believe this could be the case given the heavily means-tested nature of working-age social security in the UK, evidence suggest benefits are playing an increasingly prominent role for those towards the middle of the income distribution and less so for lower-income groups (Edmiston, 2018). Research exploiting admin-linked data in the US and Germany also shows that the most consistent predictors of under-reporting are: having higher incomes and savings, being in or closer towards the paid labour market, living in areas that have low social programme participation and living in situations that have lower likelihood of entitlement to public assistance and social transfers (Bruckmeier et al., 2014, pp. 812– 813; Meyer & Goerge, 2011). In addition, reporting a false negative is strongly associated with smaller, time-limited receipt of social assistance with evidence that ‘salience reduces reporting error, as households that are more dependent on government transfers are better reporters on average’ (Bruckmeier et al., 2019; Celhay et al., 2022, p. 3; Meyer & Goerge, 2011). Under-reporting then is more likely amongst middle-class households than has previously been recognised in mainstream poverty analysis. This is particularly important given that the concentration of social transfers and redistribution has become successively more ‘pro-rich’ moving further up the income distribution across contexts such as the US, UK, Canada and Australia (Garcia-Fuente, 2021). Failing to account for this systematically biases distributional analyses and evaluation of (welfare) state interventions.

In sum, poverty definition and measurement are often characterised as highly political and morally loaded (Lister, 2021). Less so are the infrastructure and data practices upon which conceptualisation and measurement so often depends. As demonstrated in this section, current treatment of data infrastructure tends to prioritise questions of data quality over coverage, often in specious ways that risk over-inflating low-income living standards and under-estimating the incidence and depth of poverty across high-income countries. To ensure robust analyses of poverty incidence and determinants, data quality issues must be addressed without further compromising on or exacerbating issues of poor representation of the lowest income cases. To reduce non-response (bias), population income surveys such as the FRS in the UK can learn from their international counterparts in Canada and Australia, which have deployed new online methods of data collection. Other more conventional approaches include increasing financial incentives as well as exploiting admin-linked data to correct for measurement error and ascribe ‘missing’ benefit expenditure to the correct parts of the income distribution.

## Discussion and conclusion

This article has demonstrated how dominant methods of poverty analysis currently render certain populations, and degrees of privation, more socially legible than others across high-income countries. Established practices surrounding data collection and processing rest on and reproduce structures of (mis-) recognition that make those experiencing the deepest forms of poverty less empirically and analytically visible in the wider sociocultural and political imaginary. In this way, official poverty statistics and the data practices underpinning them are both performative and normative: they describe but also ‘make’ particular social groups objects worthy of attention and intervention (Ruppert & Scheel, 2021, p. 4). A consequence (well-intentioned or otherwise) is that poverty statistics are not solely regimes of ‘knowing’, they are also regimes of ‘unknowing’ the social world, making hyper-minoritised people invisible or absent – as ‘nothing’ – in wider social scientific and distributional analyses. Here, it is worth reflecting on the ‘negative space around marked social objects’ (Scott, 2018, p. 6) that mainstream poverty analysis creates when it foregrounds particular groups on a low income, whilst re-marginalising others.

First, the inconsistent application of different measures glosses over definitional questions surrounding (deep) poverty as well as inequality below the poverty line. Current approaches demonstrate the power and pitfalls of a threshold approach to (deep) poverty measurement producing possibilities for both visibility and erasure across the social sciences. Depending on the measure applied, the extent and problem of deep poverty changes considerably, as well as the risk factors and people associated with it. By moving beyond a singular threshold indictor or aggregate measure of poverty depth, certain groups emerge as a priority in welfare politics and policy. For example, single, childless and workless households, men, ethnic minorities, young and close-to-retirement adults are all at particular risk of deep poverty in the UK, highlighting areas where targeted support would prove most effective. In the US, single, childless adults also emerge as a group worst affected by welfare state restructuring when multiple measures of poverty depth are applied across the low-income distribution. More broadly, current definitions and measures of (deep) poverty are somewhat free-floating from the living standards and social outcomes of those subject to its violence, underlining the need for an empirically informed approach to poverty measurement that captures the changing intensity of poverty and its determinants.

Second, government reporting on low incomes currently excludes distinctive, heterogeneous groups known to experience deeper forms of poverty than the wider population. At present, many hyper-marginalised groups are missing from population income surveys, undermining the accuracy of poverty estimates and public understanding of its dynamics. In contexts such as the UK and US, these data practices generate epistemic frameworks that mean the living standards of white, domiciled, ‘non-disabled’ citizens are better captured by and served through official poverty statistics, than is the case for mobile populations, ethnic minorities, migrants, and those experiencing limiting health conditions or disabilities. Much more than merely technical or practical decisions, these are theoretical and normative judgements about who counts. Failing to incorporate the non-private-household populations and their distinctive characteristics in mainstream poverty analysis risks misunderstanding the factors and causal conditions linked to (deep) poverty.

Third, shortcomings in the quality and coverage of income data across high-income countries place distributional analyses on shaky ground when researching (deep) poverty. In contexts such as the UK, a sharply declining response rate to the main household survey underpinning official poverty statistics should give considerable cause for concern. However, there is little publicly available information on the changing extent of non-response bias and how the Department for Work and Pensions has adapted its practices to account for this. Where concerns about data quality have been raised, attempts to manage uncertainty have tended to prioritise data exclusion over data manipulation in the UK. Not only is this likely to underestimate the full extent, dynamics and depth of poverty, it also risks reproducing the very exclusions implicated in (extreme) disadvantage. Leaving aside the opportunities and lessons that admin-linked data present internationally, current strategies to improve the quality of income data rationalise ways of knowing that question and peripheralise the presence of the deepest forms of poverty in high-income contexts.

To fully understand the epistemic (and ontological) significance of these data practices, examination of mainstream poverty analysis cannot be restricted to solely consider those who are currently ‘named’ or represented in distributional and social scientific analysis (Du Toit, 2009, p. 240). To mobilise effective forms of sociological analysis capable of fully apprehending the classed dynamics and social relations that make (extreme) marginality possible, a critical consideration is also needed of *whether* and *how* the social injuries of ‘the poor’ are sufficiently captured in mainstream data practices, as well as who is missing altogether. At present, most quantitative poverty analysis that attempts to make the world legible tends towards parsimony which, in turn, risks misunderstanding or misreading social relations. However, these approaches also reflect and reinforce an approach to the governance of marginality that shapes ‘fields of visibility and intelligibility within which class-based inequalities are naturalised, reproduced and legitimated’ (Tyler, 2015, p. 507). For example, Tyler (2015) demonstrates how that which is represented, designated and replicated through classificatory systems comes to animate a ‘biopolitics of disposability’ whereby already-minoritised groups are pushed further to the economic margins into poverty in highly unequal, neoliberal times. Specifically, how a selective obsession with and appraisal of disadvantage, pathologised as the result of individual behaviours and moral deficits, is central to the project(s) of neoliberal governmentality. Crucially, though, it is also the omission and absence of social phenomena in the sociocultural and political imaginary that enable deepening inequalities. As demonstrated in this article, distinctive groups and formations of privation are occluded through mainstream poverty analysis which entails the systematic ‘unseeing’ of a relatively small, but politically important population who are often worst affected in neoliberal times. It is this, as much as the sites and systems of classification, that makes a ‘biopolitics of disposability’ – and perhaps its most violent technologies – possible.

When distinctive groups are rendered less analytically and empirically legible through prevailing data practices, so are the forms of denigration and exploitation that structure their marginality. In seeking to quantify (dis)advantage, the wider adoption of these approaches across the social sciences functions to re-marginalise the privation experienced by many men, women, ethnic minorities, displaced and mobile populations, migrants, and people experiencing limiting health conditions or disabilities. The hidden forms of social violence and injustice experienced are effectively surfaced through *post hoc* surveys and qualitative research (Edin & Shaefer, 2015; Edmiston et al., 2022a; Fitzpatrick et al., 2020; Jolly et al., 2022). However, the methodological and substantive insights from such work do not always sit comfortably within or even alongside prevailing data practices and government reporting on low incomes. The failure to integrate lessons from this work significantly undermines the explanatory purchase of official statistics and distributional analysis to give the fullest account of late capitalist dynamics and its associated crises (Williams, 2021). Within mainstream poverty analysis, effacement of the gendered, racialised, bordering or ableist practices underpinning state–citizen dynamics obscures those logics and relations that are much harder to explain away or justify through a meritocratic frame of individual failings or moral turpitude. Here, we can see how the social sciences have become implicated in the reproduction of (dis) advantage, not only through ‘group-making as its technique of enquiry’, but also through the centring of ‘analytical idioms’ and explanatory frameworks that help ‘political operators to project a falsely rationalised vision of their rule’ (Wacquant, 2013, p. 277).

In this way, mainstream poverty research reveals a paradox in the public governance of marginality, where (welfare) states expend considerable amounts of money, time and attention on a number of groups that are either less visible in, or ‘missing’ from, distributional analyses. For example, those living in temporary accommodation, prisons or immigration removal centres are excluded from official poverty statistics despite being targets of considerable government intervention and *procedural* surveillance. However, the forms of government intervention they are subject to often risk reproducing marginality and dispossession (Mayblin, 2019). Making this, and the distributional outcomes associated, legible within mainstream poverty analysis, would also make the social relations and state mechanisms of devaluation subject to greater public scrutiny and accountability. Here, the social violence of parsimony in prevailing data infrastructure and practices leads to an impoverished analysis of global financialised capitalism and legitimates a biopolitics of disposability, particularly its gendered, racialised, bordering and ableist functions. What can and should be done to address this? As detailed in this article, mainstream poverty analysis urgently needs to address issues of data quality, measurement and coverage to render visible the complex dynamics that give shape and legitimation to extreme marginality. This requires the integration of hitherto siloed insights across distinct subfields to move beyond and improve upon existing approaches in a way that engenders ‘other ways of seeing and imagining poverty’ (Du Toit, 2009, p. 241).

## Figures and Tables

**Table 1:** Average incomes and rates of food insecurity at different poverty thresholds, 2019–2020.^1^

*£ per annum, net equivalised household income after housing costs* (*2019–20 prices*)	Median income	Median income (% of poverty line)	Food insecure	Severe food insecurity
All	*£*25,446	167%	8%	4%
60% of median incomes	*£*11,263	74%	21%	10%
50% of median incomes	*£*9,438	62%	23%	12%
40% of median incomes	*£*7,665	50%	23%	12%
50%+ below the poverty line	*£*5,736	38%	24%	14%

Source: DWP (2022b), author’s calculation.

**Table 2:** Poverty rates and demographic composition, 2019–2020.^2^

	Rates of poverty amongst. .				Proportion of those in different income categories that are. .			All
	60% median income	40% median incomes	50%+ below poverty line		60% median income	40% median income	50%+ below poverty line	
*Individual characteristics*								
All	22%	10%	6%					
Women	23%	10%	6%		53%	50%	49%	51%
Children	31%	13%	7%		30%	27%	23%	21%
Disability	27%	12%	7%		27%	26%	26%	22%
BAME^a^	38%	18%	12%		24%	25%	25%	14%
Asian^a^	37%	17%	11%		14%	13%	14%	8%
Black^a^	40%	21%	13%		6%	7%	6%	3%
Aged 16–24	25%	12%	7%		12%	13%	13%	10%
Aged 55–64	21%	12%	8%		12%	15%	17%	13%
Aged 65+	19%	6%	4%		15%	11%	11%	18%
*Household composition* ^b^								
Larger families	45%	18%	8%		7%	6%	4%	3%
Workless household	33%	17%	11%		56%	58%	59%	36%
Single male workless	47%	31%	23%		14%	20%	22%	7%
Single female workless	47%	27%	18%		15%	18%	18%	7%
Lone-parent family, workless	62%	33%	18%		5%	5%	5%	2%
Couple with children, part-time work	63%	32%	18%		3%	3%	3%	1%

Source: DWP (2022b), author’s calculation.

a Figures based on three-year averages (2017–2020).

b Household composition refers to characteristics of the ‘benefit unit’ and are weighted accordingly.

**Table 3:** Income and characteristics of people across the income distribution, 2019–2020.

	Median income^a^	Mean income^a^	Food insecure	Female	BAME	Private renter	Receiving benefits^c^	Self- employed^c^
Bottom 3 income percentiles^a^	–*£*14	–*£*32	17%	49%	24%	38%	24%	14%
In relative poverty^b^	*£*203	*£*173	21%	53%	24%	29%	46%	8%
All	*£*476	*£*587	8%	51%	15%	19%	20%	8%

Source: DWP (2022b), author’s calculation.

a £ per week, net equivalised household income after housing costs (2019–2020 prices).

b Those falling more than 60% below median incomes (after housing costs).

c One or more person in the household, working-age population.

## Notes

1 Due to issues surrounding data collection during the COVID-19 pandemic, data from 2019–2020 are drawn upon here. Due to uncertainty surrounding data quality, the bottom 3% of the income distribution have also been excluded from these figures but the risks associated with this are discussed later.

2 See note 1.

3 Figures for the UK are derived from national censuses of England and Wales. In addition to the private household population typically sampled in household income surveys, censuses also cover the non-private-household population, such as those residing in communal establishments and temporary accommodation (Bramley et al., 2018, p. 17).

4 Indeed, previous work has sought to better account for the non-private-household population in sampling strategies and analysis (e.g. Townsend, 1979, pp. 696, 939–944).

5 The central government department responsible for reporting on low incomes in the UK.

6 During the first year of the pandemic, the response rate to FRS fell to 23% given the extraordinary challenges surrounding data collection at the height of COVID-19 and is yet to return to pre-pandemic levels.

7 It has, of course, also been used to consider the functions of different measures of material privation in terms of what these can (and cannot) tell us about the nature of poverty, those at greatest risk of it and trends over time (Hick, 2015).

8 The current approach to data cleaning, imputation and use of administrative records in the FRS is broadly outlined by the Department for Work and Pensions but it remains to be seen how integrated survey-administrative datasets will be used in the future (DWP, 2022c).
